# The Dental Law in Ohio

**Published:** 1878-11

**Authors:** 


### Editorial.

### THE DENTAL LAW IN OHIO.

Occasionally complaints are made that persons are practicing without having conformed to the requirements of the law, made and provided, for the regulation of the practice of dentistry in Ohio. And usually accompanying such complaint is the question, "What ought, and what can be done?"

To the first part of the question there is but one answer, viz: Every one attempting to practice dentistry in Ohio should conform to the requirements of the law, and if he neglects it, he should receive adequate attention and assistance.

This should be done for several reasons:

The open and persistent violation of good and wholesome law is always demoralizing.

The best interests of the public require that conformity to this law should be observed and enforced. It gives to the people a standard by which they may, somewhat at least, judge of the qualifications of those who practice in this important branch of surgery and medicine; and such a standard, and better if possible, they should have in all cases. It is said that the people are careless in this matter. Of some that may be true, but it is not of all nor the majority, and the number of such careless ones is constantly and surely becoming less, as education and culture increase.

The best interests of the dental profession require an entire conformity to this law. It stimulates to higher attainments, and greater efficiency, those who seek to enter its practice; and as one attains these he far better realizes the dutv and responsibility that devolve upon him professionally, and this apprehension gives strength and efficiency to all he does.

The true, efficient and responsible part of the profession has borne a great dead weight—carried a great incubus in the form of incompetency, assumption and charlatanry.

Previous to the enactment of this law in Ohio, not more than one in five of those practicing dentistry had any adequate fitness for the work; many of them lacking natural endowment, and a far larger proportion, educational fitness; and too often in the popular estimate this class was the criticism by which all were judged. Such embarrassments are demoralizing and discouraging. The efforts for developing and strengthening the profession have been engaged in by comparatively few members of the profession, and these have labored under difficulties, many of which ought not to exist, and would not were there more extended co-operation.

A strict conformity to the law would add great encouragment, strength and power for good to the profession in the State, and indeed would not be without its influence outside of the State.

In reply to the complaint that the law is not obeyed or enforced, which is doubtless true in some, perhaps many, instances, it may, we think, with entire correctness be said, that it is as well regarded aad obeyed as a large majority of the laws of the State, and much better than some laws that have a more extensive outreach than this.

It is not by the number of violations of a law and punishment for the same that its value and efficiency is to be estimated, but by the general acceptance of, and acquiescence in its requirements and regulations on the part of those upon whom it is operative.

There are in Ohio about five hundred and eighty dentists. About two hundred of these have complied with the law by passing the required examination before the State Board of Examiners; about one hundred and eighty are regular graduates, leaving about two hundred who are neither graduates nor have certificates of qualification by examination.

All who had been in practice in the State five years and over at the time of the passage of the law as amended in April, 1873, were, by its provisions, exempt from its requirements. There were probably nearly, if not quite, two hundred of the latter class in practice in the State at the time of the passage of the law. A somewhat extended observation fully warrants this statement. There are very few dentists practicing in this State in violation of law. A few there are doubtless who have steathily crept in, one here and another there, who are in practice illegally, but the fact that they do so, is in no respect chargable to the law. Many good laws are openly violated daily in every city, village and hamlet in the land, and yet how few are there who exercise any active effort to have offenders punished.

The dental law has wrought great good for the public and for the profession in the State. It has given to the public a much larger proportion of efficient dentists than there ever was before. There has probably been more students from Ohio than from any other state, in the dental colleges of the country, and many who have remained at home have applied themselves more industrously than ever before in the direction of better preparation.

The question is sometimes asked, "How can a better observance of the law be secured?"

In reply to this it may be said that it is the duty of every good citizen to do what he can to secure obedience to every just and wholesome law; this is true of the dentist as well as of any one else, and especially should he have a warm interest in the observance of this law, because it affects his professional life and action.

But one says, what can I do? There are several things you can do. You can go to an offending member and give him to understand clearly that he is violating a good law, one for the public welfare, and for the benefit of the profession as well. One in which there is full and hearty acquiesence on the part of the profession in the State with only here and there an exception; that it ought to be obeyed by all; that it would be better for the whole, were it so; that no one can afford to place himself in a state of isolation from, much less in a state of antagonism to the profession; that the best interests of each one lies right along in the channel with the great body of the profession.

If, after such a presentation, there is still resistance, let several persons jointly present the matter, and ultimately make it clear that obedience to the law must be rendered; and very rarely, if ever, will any thing further be required. But if after all this there is still resistence and violation of the law, then let the profession of the locality in question co-operate for securing obedience, even though the law's penalties must come in as an argument. The procedure in such a case is very simple. First obtain the facts and sufficient proof, then bring the matter to the notice of the prosecuting attorney; he must then take the case to the grand jury, and if there is good proof, an indictment will be found; the case then goes before the court for trial and disposal.

The dentists of Ohio owe it to the profession of which they are members, and each to himself individually, to see to it, that this law is strictly obeyed by every practitioner in the State.

